# Three-year evaluation of the nosocomial infections in pediatrics: bacterial and fungal profile and antimicrobial resistance pattern

**DOI:** 10.1186/s12941-022-00496-5

**Published:** 2022-02-16

**Authors:** Mehrnoush Afsharipour, Shima Mahmoudi, Hojatollahh Raji, Babak Pourakbari, Setareh Mamishi

**Affiliations:** 1 Pediatrics Center of Excellence, Children’s Medical Center, Tehran University of Medical Sciences, Tehran, Iran; 2 Pediatric Infectious Disease Research Center, Tehran University of Medical Sciences, Tehran, Iran; 3 Department of Pediatric Surgery, Pediatrics Center of Excellence, Children’s Medical Center, Tehran University of Medical Sciences, Tehran, Iran; 4 Pediatric Infectious Disease Research Center, Pediatrics Center of Excellence, Children’s Medical Center Hospital, Dr. Gharib Street, Keshavarz Boulevard, Tehran, Iran

**Keywords:** Nosocomial infections, Antimicrobial susceptibility, Pediatrics

## Abstract

**Background:**

Nosocomial infections (NIs) could lead to considerably higher mortality rates, length of the hospital stays and costs, and represent a serious public health concern worldwide. Besides, the unreasonable use of antibiotics could lead to get resistant to different antibiotics and create limited therapeutic options, increased risks of treatment failure and poor patient management. The current study aimed to evaluate the prevalence and antimicrobial susceptibility of NIs in an Iranian referral pediatrics hospital during 3 years.

**Methods:**

During the 3-year period, all electronic medical records of nosocomial infection episodes in hospitalized patients were retrospectively reviewed. The bacterial and fungal profile and antimicrobial susceptibility profiles of isolates recovered from different samples of patients with NIs were determined.

**Results:**

In this study, a total of 718 patients with NIs was found, among which 61.3% were male (N = 440). The median age of the patients was 2.5 years (IQR: 1 month to 3 years). *Klebsiella pneumonia* and *Candida* spp. isolates were the most prevalent microorganisms (N = 125, 17.4%, N = 121, 16.9%, respectively), followed by *Pseudomonas aeruginosa* (N = 72, 10%) and *Coagulase-negative Staphylococci* (CoNS) (N = 69, 9.6%). *Pseudomonas aeroginusa* strains showed high sensitivity to the studied antibiotics. *Acinetobacter baumannii* strains displayed more than 90% resistance to the almost all antibiotics. All of the tested isolates of *S. maltophilia* were susceptible to Trimethoprim−sulfamethoxazole (100%) and showed high susceptibility rate to ciprofloxacin (96.4%). Vancomycin resistance was not reported in *S. aureus* isolates, while 64% of *Enterococcus * spp. was resistant to vancomycin. The rates of methicillin resistance for *S. aureus* and CoNS isolates were 45.5% and 85.7%, respectively.

**Conclusions:**

High frequency of antimicrobial resistance to the commonly tested antibiotics is a concerning alarm. Therefore, effective infection control programs and rational antibiotic use policies should be established promptly.

## Introduction

A nosocomial infection (NI) (also known as hospital-acquired infection) is a localized or a systemic infection occurring with an adverse reaction to infectious agents that develops in 48 h or more after admission [1]. NIs could lead to considerably higher mortality rates, length of the hospital stay and costs, and represent a serious public health concern worldwide [2, 3]. The leading bacteria related to NIs are *Staphylococcus aureus*, coagulase-negative staphylococci (CoNS), *Streptococcus pneumoniae*, *Escherichia coli*, *Pseudomonas aeruginosa, Haemophilus influenzae, Klebsiella pneumoniae, Acinetobacter*, and *Enterococci* [4, 5].

Nowadays, antibiotics remain the leading therapy for treating bacterial infections. However, by the unreasonable use of antibiotics, certain strains of multidrug-resistant (MDR) bacteria have emerged by selection pressure; consequently, bacteria that have been once sensitive, re-emerged as resistant to different antibiotics and create limited therapeutic options, increased risks of treatment failure and poor patient management [6]. Knowledge of proper antimicrobial prescription policy of a particular setting in addition to the investigation of causative agents and their antimicrobial susceptibility profile, is essential to improve the management and reduction of the rate of NIs [7]. The aim of the current study was the evaluation of the frequency and antimicrobial susceptibility of NIs in an Iranian children medical center during three years.

## Materials and methods

This cross-sectional study was carried out in the referral hospital of Children’s Medical Center, Tehran, Iran between March 2017 and February 2020. Ethical approval (IR.TUMS.CHMC.REC.1399.037) was obtained from the Ethical Committee of Tehran University of Medical Sciences, Tehran, Iran.

All patients who admitted to the medical wards of Children’s Medical Center, Tehran, Iran for more than 48 h and had the evidence of NIs with positive blood, wounds and sterile fluids culture of gram-positive/gram-negative bacteria and fungi were included in this study. Duplicate isolates from one patient were excluded from the study. In vitro phenotypic characterization of bacteria or fungi was carried out using standard culture and biochemical tests as described previously [8]. The disk diffusion method or minimal inhibitory concentration (MIC) was used to test each isolate for in vitro antimicrobial susceptibility based on the Clinical and Laboratory Standards Institute criteria [9].

The following antibiotics disks from MAST Categories Ltd., Merseyside, UK, were used: imipenem (10 µg), ampicillin (10 µg), cefotaxime (30 µg), clindamycin (2 µg), Trimethoprim−sulfamethoxazole (1.25/23.75 mg), ceftazidime (30 µg), nitrofurantoin (200 µg), ceftriaxone (30 µg), erythromycin (15 µg), gentamycin (10 µg), cefepime (30 µg), penicillin (10 µg), linezolid (30 µg), cefoxitin (30 µg). *Staphylococcus aureus* ATCC 25,923 was used for quality control of the test. The MICs of vancomycin and colistin were determined by E-test methods.

Statistical analysis of the results was performed using SPSS 13.0 (SPSS Inc. Chicago, IL, USA). The results were presented as mean, frequency and standard deviation for quantitative and percentage and frequency for qualitative data.

## Results

In the current study, a total of 718 patients included, among which 61.3% were male (N = 440). The median age of the patients was 2.5 years (IQR: 1 month–3 years). Among the patients, 27.2% had underlying heart disease (N = 195) and 16.3% had seizures (N = 117). Intrinsic and acquired immunodeficiency was also reported in a number of patients (N = 35, 4.9%, N = 59, 8.2%, respectively). Three hundred and eighty-four patients (53.5%) utilized catheters, and 101 of them (14.1%) had endotracheal tube during their hospitalization.

The frequency of isolated microorganisms among the studied patients based on the sources of their isolation was mentioned in Table 1. *Klebsiella pneumonia* and *Candida *spp*.* were the most prevalent isolates (N = 125, 17.4%, N = 121, 16.9%, respectively), followed by *P. aeruginosa* (N = 72, 10%) and CoNS (N = 69, 9.6%). Also, most of the samples were isolated from blood (N = 495, 69%), followed by sterile fluids (N = 165, 23%) and finally wounds (N = 58, 8%). *Klebsiella pneumonia* was the most frequent organism isolated from blood and wounds, and *Candida *spp*.* was the most frequent organism isolated from sterile fluids.

**Table 1:** The frequency of isolated microorganisms among the studied patients

Bacteria	Blood [N (%)]	Sterile fluids [N (%)]	Wound [N (%)]	Total [N (%)]
*K. pneumonia*	84 (16.9)	26 (15.8)	15 (25.9)	125 (17.4)
*Candida* spp.	81 (16.4)	34 (20.6)	6 (10.3)	121 (16.9)
*P. aureoginosa*	39 (7.9)	24 (14.5)	9 (15.5)	72 (10)
*CoNS*	62 (12.5)	4 (2.42)	3 (5.2)	69 (9.6)
*Acinetobacter baumannii*	18 (3.6)	29 (17.6)	4 (6.9)	51 (7.1)
*Psuedomonas* spp.	39 (7.9)	9 (5.45)	0	48 (6.7)
*Enterococcus* spp.	39 (7.9)	2 (1.21)	6 (10.3)	47 (6.5)
*E. coli*	25 (5)	9 (5.45)	7 (12.1)	41 (5.7)
*S. aureus*	28 (5.7)	3 (1.82)	4 (6.9)	35 (4.9)
*Serratia marcescens*	24 (4.8)	5 (3.03)	2 (3.4)	31 (4.3)
*Stenotrophomonas maltophilia*	24 (4.8)	6 (3.6)	1 (1.72)	31 (4.3)
*Enterobacter* spp.	19 (3.8)	8 (4.8)	1 (1.72)	28 (3.9)
*Streptococcus* spp.	6 (1.2)	2 (1.2)	0	8 (1.1)
*Burkholderia cepacia*	3 (0.6)	3 (1.8)	0	6 (0.8)
*Morganella morganii*	2 (0.4)	0	0	2 (0.3)
*Aspergilus* spp.	0	1 (0.6)	0	1 (0.1)
*Salmonella* spp.	1 (0.2)	0	0	1 (0.1)
*Haemophilus* spp.	1 (0.2)	0	0	1 (0.1)
Total	495 (100)	165 (100)	58 (100)	718 (100)

There was a slight decrease in the total number of isolates each year compared to the previous year (the first year: 272 patients, 37.9%; the second year: 234 patients, 32.6%; the third year: 212 patients, 29.5%). *Morganella morganii* and *Haemophilus* spp. specimens were isolated only in the first year of the study (2017). During these years, *Serratia marcescens* (n = 13, n = 12, n = 6, respectively) and *S. aureus* (n = 19, n = 10, n = 6, respectively) showed a decreasing trend. While the *Enterococcus* spp. (n = 21, n = 13, n = 13, respectively) and *P. aeruginosa* (n = 30, n = 21, n = 21, respectively) after a 2-year downward trend, in 2019, remained stable. The frequency of *Pseudomonas* spp. (n = 6, n = 17, n = 25, respectively) and *Enterobacter* spp. (n = 5, n = 16, n = 17, respectively) represented an increasing trend.

Most of the isolates were collected from hospitalized patients at neonatal intensive care unit (NICU) and pediatric intensive care unit (PICU) (N = 109, 15.2%, N = 100, 13.9%, respectively) and the most isolated microorganisms from them were *K. pneumonia* (N = 29, 26.6%) and *Candida* spp. (N = 25, 25%), respectively.

Antibiotic susceptibility frequencies of evaluated microorganisms were depicted in Table 2. *Escherichia coli*,* Acinetobacter baumannii*,* S. marcescens*,* K. pneumonia* and* Pseudomonas* spp. strains showed 100% sensitivity to colistin. *Pseudomonas aeroginusa* strains as a whole showed significant sensitivity to the studied and the most sensitive antibiotics were imipenem (80.4%) and ceftazidime (80.8%). Subsequently, the highest sensitivity to ceftazidime was observed in *Pseudomonas * spp. (79.2%), while *A. baumannii* strains showed 94.8% resistance to this antibiotic.

**Table 2:** The percentage of antimicrobial susceptibility of NIs in an Iranian referral pediatrics hospital

Bacteria	Gentamycin	Trimethoprim sulfamethoxazole	Imipenem	Nitrofourantoin	Cefotaxime	Clindamycin	Ampicillin	Ceftazidim	Penicillin
*E. coli*	58.6%	16.7%	22.2%	100%	12.5%	–	0%	33.3%	22.7%
*Enterococcus *spp.	23.5%	100%	50%	88.9%	0%	0%	26.9%	–	–
*Psuedomonas *spp.	23.8%	–	84.2%	–	–	–	–	79.2%	–
*P. aureoginosa*	72.7%	75%	80.4%	–	62.5%	–	–	80.8%	–
*K. pneumonia*	37.5%	37.2%	52.9%	33.3%	4.4%	100%	100%	100%	–
*CONS*	0%	35.4%	–	50%	0%	18.6%	0%	–	0%
*S. aureus*	0%	78.3%	100%	100%	–	45.5%	–	–	14.3%
*Enterobacter *spp.	50%	36.4%	100%	0%	14.3%	–	0%	100%	0%
*Acinetobacter baumannii*	23.7%	9.4%	5.3%	–	2.4%	–	16.7%	5.3%	–
*Serratia marcescens*	13.6%	100%	100%	–	16%	–	–	100%	–
*Stenotrophomonas maltophilia*	0%	100%	0%	–	–	–	–	–	0%
*Candida *spp.	–	–	–	–	–	–	–	–	
*Streptococcus *spp.	–	0%	–	–	–	50%	100%	–	100%
*Burkholderia cepacia*	33.3%	100%	33.3%	0%	100%	–	0%	66.7%	–
*Morganella morganii*	50%	100%	–	–	100%	–	–	–	–
Total	41.1%	48.4%	59.4%	63.8%	10.1%	29.6%	29.5%	56.5%	12.6%

Bacteria	Vancomycin	Colistin	Methicillin	Erythromycin	Ciprofloxacin	Tazocin	Linezolide	Amikacin	Cefepime
*E. coli*	–	100%	–	–	50.00%	59.3%	–	78.1%	17.9%
*Enterococcus *spp.	36%	–	–	0%	0%	40%	100%	60%	66.7%
*Psuedomonas *spp.	–	100%	–	–	100%	90%	–	34.8%	50%
*P. aureoginosa*	100%	50%	–	–	75%	78.4%	–	81.8%	62%
*K. pneumonia*	100%	100%	–	–	55.6%	23.8%	–	25.5%	11.5%
*CONS*	100%	–	14.3%	4.5%	–	100%	–	50%	–
*S. aureus*	100%	–	54.5%	35%	0%	–	–	–	–
*Enterobacter *spp.	0.00%	–	–	–	33.3%	68.4%	100%	73.7%	47.1%
*Acinetobacter baumannii*	–	100%	–	–	10%	9.4%	–	9.8%	5.6%
*Serratia marcescens*	–	100%	–	–	33.3%	10%	–	24%	0%
*Stenotrophomonas maltophilia*	–	–	–	100%	96.4%	–	–	0%	–
*Candida *spp.	100%	–	–	–	–	–	–	–	–
*Streptococcus *spp.	100%	–	–	0%	–	–	–	–	–
*Burkholderia cepacia*	–	0%	–	–	50%	66.7%	–	0%	0%
*Morganella morganii*	–	–	–	–	–	100%	100%	100%	100%
Total	85.2%	96.9%	28.1%	14.7%	49.4%	44.8%	100%	43.0%	26.8%

Vancomycin resistance was not reported among *S. aureus* isolates in this study. Clindamycin had the least effect on CoNS strains (18.6%). *Staphylococcus aureus* strains were highly resistant to gentamycin (100%), ciprofloxacin (100%) and penicillin (85.7%). Methicillin-resistant *S*.* aureus* (MRSA) was found in 45.5% of the isolates. However, next to vancomycin, nitrofourantoin and imipenem (each n = 1/1, 100%), and Trimethoprim−sulfamethoxazole (n = 18/23, 78.3) were the most effective antimicrobial agents on it.

High levels of resistance to gentamycin were also showed among *S. marcescens* (n = 19/22, 86.4%), *Enterococcus* spp. (n = 13/17, 76.5%), *A. baumannii* (n = 29/38), 76.3%), and *Pseudomonas* spp. (n = 16/21, 76.2%) strains.

All of the tested isolates of* Streptococcus* spp. were 100% sensitive to ampicillin and penicillin (each n = 3/3), and vancomycin (n = 4/4), but fully resistant to erythromycin (n = 2/2) and Trimethoprim−sulfamethoxazole (n = 1/1).

*Escherichia coli* showed a high level of resistance to cefotaxime (n = 28/33, 87.5%), Trimethoprim−sulfamethoxazole (n = 25/30, 83.3%), cefepime (n = 23/28, 82.2%), and imipenem (n = 7/9, 77.8%), but 100% sensitivity to nitrofourantoin (n = 14/14).

*Acinetobacter baumannii* strains also displayed more than 90% resistance to the almost all antibiotics studied including imipenem, cefepime, Trimethoprim−sulfamethoxazole, meropenem, piperacillin/ tazobactam, amikacin, ciprofloxacin, and cefotaxime. Likewise, *K. pneumonia* (n = 64/84, 76.2%) and *S. marcescens* (n = 18/20, 90%) strains were resistant to piperacillin/ tazobactam. However, this antibiotic was mostly effective on *Pseudomonas *spp*.* (n = 18/20, 90%).

All of the tested isolates of *S. maltophilia* were susceptible to Trimethoprim−sulfamethoxazole (n = 29/29, 100%) and showed high susceptibility rate to ciprofloxacin (n = 27/28, 96.4%). The isolates of Enterobacter spp*.* showed 73.7% sensitivity to amikacin (n = 14/19).

## Discussion

In this study, we evaluated the microorganisms isolated from NIs over three consecutive years which generally had a slow decreasing trend.

The present study showed *K. pneumoniae* (N = 125, 17.4%), *Candida* spp. (N = 121, 16.9%), and *P. aeruginosa* (N = 72, 10%) as the most frequent microorganisms which cause NIs among the studied children. Of course other frequent NI-causing bacteria were reported in our study including CoNS (9.6%), *A. baumannii* (7.1%), *Psuedomonas* spp. (6.7%), and *Enterococcus* spp. (6.5%). 61% of isolated organisms were gram-negative bacteria, which was about three times more than the number of gram-positive bacteria isolated in our study (22.1%).

Likewise, high rate of gram-negative bacteria was reported in Feleke et al. (53.2%) study [6]. Also unlike the study of Feleke et al. and the one reported from Jimma which mentioned *S. aureus* and *E. coli* as their the most common isolates [6, 10], in the present study, *K. pneumoniae* was the most common bacteria isolated. Similarly, in the study accomplished by Mahmoudi et al., [14] *K. pneumoniae* (n = 263, 27.5%) was reported as the most frequent bacteria. In a study by Bouza et al. [11], *E. coli* (35.3%) was the most commonly isolated microorganism, and *Klebsiella spp.* were reported as 9.8% of the pathogens. Gupta et al. [12] reported that *S. aureus* and *CoNS* as the most common isolated gram-positive bacteria which is in line with our results. Nouri et al. reported the high prevalence of gram-negative bacteria (77.9%) in NIs and low prevalence of gram-positive bacteria (22.1%), exactly as ours, and the most common bacterium causing NIs among the latter was *S. aureus* [13].

67% of isolated strains was from ICUs (mostly NICU and PICU) (N = 482), which was compatible with the results of our previous study [14]. Also, Alvares et al. reported nosocomial pneumonia as the third most common NI in their pediatric intensive care unit [2]. *Candida* spp. strains were isolated frequently from PICU (25%) and emergency ICU (24.4%). Surgical and ICU patients are at higher risk of rising nosocomial fungal infections [15]. In critically ill patients, the disseminated candida infections are the principal causes of morbidity and mortality both in immunocompetent and immunocompromised patients [16].

*A. baumannii* strains were considerably resistant to almost all tested antibiotics except for colistin (100% sensitivity), which is similar to previous studies [17, 18]. Sohail et al. [19] also showed that only 0.1% of the isolated strains were resistant to colistin. The results of study reported by Vahdani et al. [20] showed antibiotic-resistant *A. Baumannii* infections with high resistant rate to ceftazidime (96%), followed by ceftizoxime (95%), ceftriaxone (93%), ciprofloxacin (85%), and trimethoprim/sulfamethoxazole (85%). Along with the significance of MDR *A. baumannii* in NIs, the increasing reports of outbreaks caused by carbapenem-resistant *A. baumannii* in recent years have become another frightening reality [21].

In the present study, *K. pneumonia* strains were highly resistant to cefotaxime (95.6%), while showed 100% susceptibility to colistin, vancomycin, ampicillin, ceftazidime and clindamycin. Sensitivity to gentamycin reported as low as 37.5% among *K. pneumonia* strains in our study. Compared with the results of the study by Ares et al. [22], the resistance rates of isolates in the current study against studied antibiotics, especially carbapenems, were considerably high. This difference in the resistance patterns of *K. pneumoniae* could be due to the different prevalent clones in Iran and other countries in addition to differences in antibiotic treatment regimens in different areas [23].

All *E. coli* isolates tested in this study were sensitive to nitrofurantoin and colistin, while showing significant resistance to the other antibiotics compared to our previous study [17]. However, the resistance of this microorganism to imipenem (77.8% in comparison with 8%) has increased significantly compared to the mentioned study. High resistance to ampicillin has been reported in other studies, as well [24–26].

The frequency of MRSA (43%) was more than the amount reported by our previous study (26%) [27], Nigussie et al. and Latif et al. (38.5% and 31.25%, respectively) [28, 29].

In this study, *P. aeruginosa* strains were highly sensitive to amikacin (81.8%), imipenem (80.4%), piperacillin/tazobactam (78.4%), Trimethoprim−sulfamethoxazole and ciprofloxacin (75%). However, resistance rates of *P. aeruginosa* to gentamicin (27.3%), amikacin (18.2%) and ceftazidime (19.2%) were higher than our recent study [14]. In addition, lower resistance rate for cefepime was reported by Larru et al. (4.3%) [30] and Ares et al. (8.5%) [22], compared to the percentage of 38% in the current study.

There are only a limited number of studies describing the *S. maltophilia* infection in children [31]. Treatment of nosocomial *S. maltophilia* infections is complicated due to high rates of antibiotic resistance [32]. We reported 100% resistant *S. maltophilia* isolate to gentamycin, imipenem, and penicillin (n = 1/1). However, treatment of *S. maltophilia* infection is difficult due to antimicrobial resistance to a variety of agents; trimethoprim-sulfamethoxazol can continue to be the first choice for the treatment of *S. maltophilia.* In the study performed by Alsuhaibani et al., [33] the most effective antibiotic against *S. maltophilia* isolates was Trimethoprim−sulfamethoxazole (94.1%), which is consistent with our data (100%). Also in the study by Sun et al. [32], the resistance rate of *S. maltophilia* strains to cefpime, cefotaxime, ceftazidime and gentamicin was 45.1%, 94.1%, 60.8% and 82.4%, respectively.

Regarding the frequency of resistance to vancomycin, no cases were reported among *S. aureus*, while 64% of *Enterococcus* spp. were resistant to vancomycin that is similar to our recent previous study [27] and is higher than our previous studies in our hospital during 2009–2010 [34]. Since NIs are an important determinant in hospital, improving of the prevention and treatment of NIs is still highly needed [7].

## Conclusions

High frequency of antimicrobial resistance to the commonly tested antibiotics is a concerning alarm. Therefore, effective infection control programs and rational antibiotic use policies should be established promptly.

## Data Availability

All data obtained.

## Abbreviations

NIs: Nosocomial infections
CoNS: Coagulase-negative staphylococci
MDR: Multidrug-resistant
MIC: Minimal inhibitory concentration
NICU: Neonatal intensive care unit
PICU: Pediatric intensive care unit
